# Increased temperature selects for antibiotic resistance genes in a model aquatic ecosystem

**DOI:** 10.1038/s44259-026-00231-6

**Published:** 2026-06-12

**Authors:** Emma Bousquet, Sabine Favre-Bonté, Thibault Meyer, Concepción Sánchez-Cid

**Affiliations:** Université Lyon 1, CNRS, INRAE, LEM, UMR 5557, UMR 1418, Villeurbanne, France

**Keywords:** Ecology, Ecology, Environmental sciences, Microbiology, Molecular biology

## Abstract

There is a rising concern that temperature increases associated with climate change might select for antibiotic-resistant bacteria (ARB) and antibiotic resistance genes (ARGs) in aquatic ecosystems, although the extent of this phenomenon remains unknown. This study aimed to determine whether increased temperatures induce ARG selection and modify the transcriptional response in a model aquatic ecosystem using an omics approach. River water microcosms were incubated at 20 °C, at 28 °C (constant temperature increase), and under oscillating temperatures (intervals of 3 days at 20 °C and 4 days at 28 °C to emulate heatwaves) for 28 days. Both the constantly higher temperature and oscillating temperatures altered bacterial community composition and selected for members carrying ARGs, including two *Limnohabitans* subpopulations that contain the *pmrE* polymyxin resistance gene and an Alphaproteobacteria carrying the *sul2* sulfonamide resistance gene. In addition, metatranscriptomic analyses revealed a lower abundance (*p* < 0.05) of transcripts related to cell division, bacterial activity/metabolism, antibiotic efflux, stress responses, and cell/gene mobility in samples exposed to oscillating temperatures than in the room temperature controls. This research supports the rising concern that climate change may increase antibiotic resistance in environments exposed to higher temperatures maintained over long periods of time and to repeated short-time heatwave events.

## Introduction

The antibiotic resistance crisis is a global environmental and public health concern. Understanding the drivers of antibiotic resistance dissemination in environmental settings is a critical aspect to the management of the antibiotic crisis^1^. Aquatic ecosystems are at the interface of anthropogenic input from multiple sources^2^ and they are subjected to a wide variety of environmental and anthropogenic stresses that may promote antibiotic resistance selection and dissemination^3^. Recently, the global increase of average temperatures and extreme temperature fluctuations has been suggested as a potential selective pressure that might contribute to antibiotic resistance spread^4–8^. Increases in antibiotic resistance in human pathogens have been associated with higher local temperatures^9,10^. In addition, temperature influences the composition, activity, and functioning of complex bacterial communities^11,12^. Furthermore, stress response mechanisms known to be involved in antibiotic resistance could be involved in the response to thermal disturbance^13^. Specifically, responses to heat stress and antibiotics overlap in *E. coli*^14^, and the overexpression of chaperones involved in the heat shock response protected *E. coli* from sub-lethal aminoglycoside pressure^15^. Finally, higher temperatures may increase horizontal gene transfer frequency^16,17^ and therefore, antibiotic resistance spread within the community. Thus, increased temperatures might accelerate the development and dissemination of antibiotic resistance. However, the extent to which temperature increases linked to climate change may affect antibiotic resistance in aquatic ecosystems remains unknown.

The goal of this study was to evaluate the impact of increased water temperatures on bacterial communities from river water and their associated antibiotic resistance genes. The Rhône River was used as an aquatic ecosystem model since it is one of the largest rivers in Europe: it originates in the Alps and flows through several climate zones in Switzerland and France (including large cities such as Geneva and Lyon) before its discharge into the Mediterranean Sea. Two different types of temperature increases were tested: a constant increase aimed at representing the rise of mean temperatures and a series of oscillating increases aimed at emulating short-term heatwave events. We hypothesized that increased temperatures select for non-sensitive bacteria, indirectly selecting for antibiotic resistance genes harbored by these populations. Using a combination of metagenomics and metatranscriptomics, the response of aquatic bacteria to increased temperatures and the impact of thermal disturbance on antibiotic resistance gene selection in aquatic ecosystems was assessed. This study fills a critical knowledge gap regarding the impact of prolonged exposure to increased temperatures and repeated short-term temperature increases on microbial ecology in riverine ecosystems.

## Results

### Increased temperature affects the composition of bacterial communities and the antibiotic resistome

**Fig. 1:**
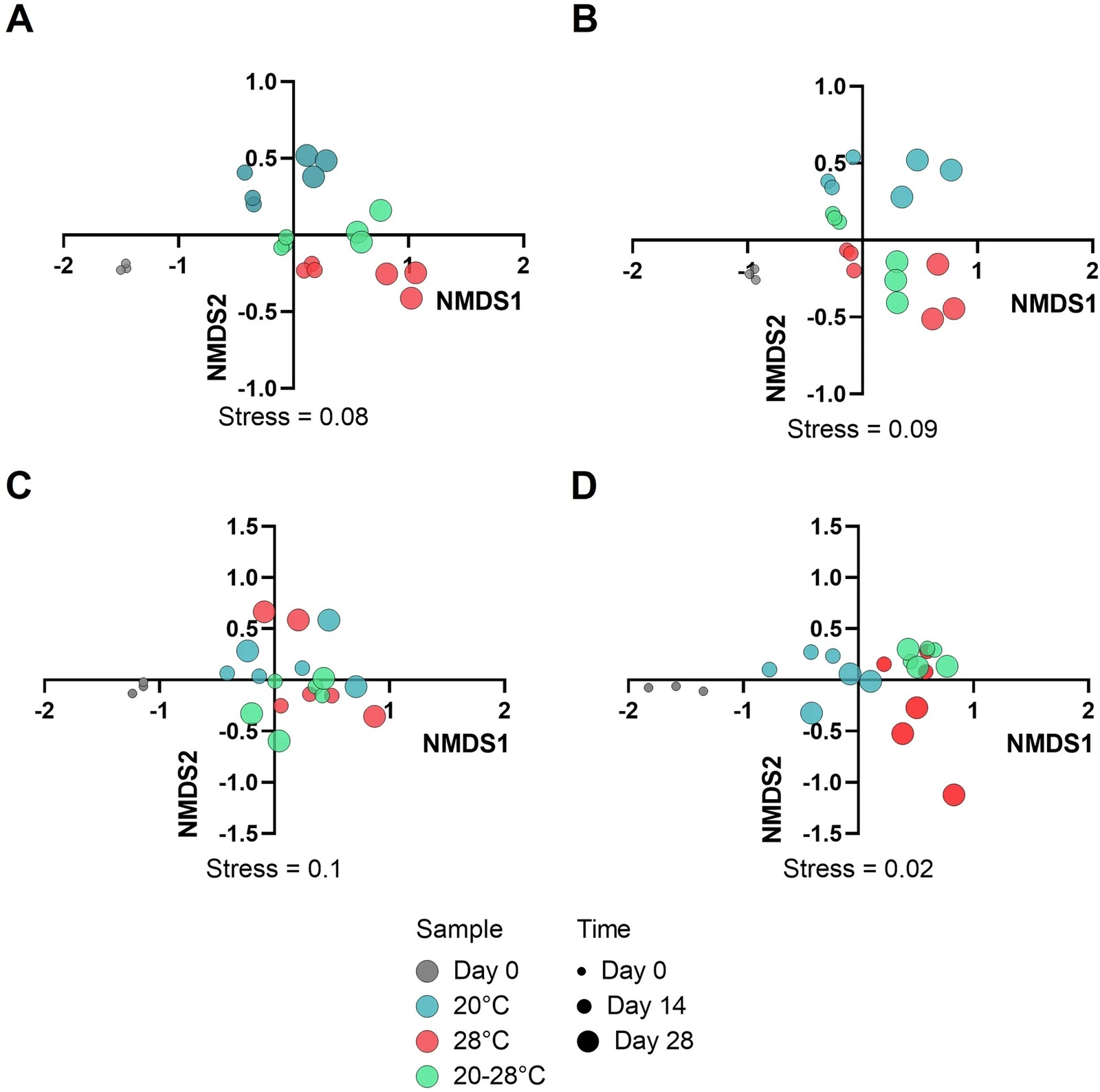
Impact of increased temperature on overall bacterial community and resistome composition. Non-metric multidimensional scaling of the Bray-Curtis distances calculated from the relative abundance of all metagenome assembled genomes (MAGs) obtained from the metagenomic sequencing (**A**), MAGs containing antibiotic resistance genes (ARGs) (**B**), contigs containing ARGs involved in antibiotic efflux (**C**) and contigs containing all other ARGs (**D**). Relative abundances were calculated using the percentage of recruitment of MAGs and contigs across samples, which represents the percentage of sequences from a sample recruited into a MAG or a contig. PERMANOVA analysis on temperature impact without day 0: all MAGs R^2^ = 0.33 F = 10 *p* = 0.001; ARG-containing MAGs R^2^ = 0.31 F = 7 *p* = 0.001; contigs containing genes involved in antibiotic efflux R^2^ = 0.17 F = 2 *p* = 0.04; contigs containing ARGs R^2^ = 0.54 F = 15 *p* = 0.001. 20 °C = room temperature controls (blue). 28 °C = constant temperature increase (red). 20–28 °C = repeated temperature increases (green). *n* = 3.

Both a higher constant temperature and an oscillating temperature affected the overall composition of bacterial communities in Rhône river microcosms after 14 and 28 days (Fig. 1A) without reducing overall bacterial community abundance and transcriptional activity (Supplementary Fig. 1 in Supplementary Information). Furthermore, there was a shift in the composition of the communities (metagenome assembled genomes-MAGs) that contained ARGs in samples exposed to both a constant higher temperature and oscillating temperatures compared to room temperature controls **(**Fig. 1B**)**. In addition to the changes in the overall composition of total and ARG-containing bacterial communities, a contig-based analysis was used to determine the impact of temperature increases on the composition of the resistome and mobilome. Whereas temperature increases had a limited impact on the distribution of contigs containing genes involved in antibiotic efflux **(**Fig. 1C**)**, metal resistance genes (Supplementary Fig. 2A in Supplementary Information) and mobile genetic elements (Supplementary Fig. 2B in Supplementary Information), a shift in the antibiotic resistome was observed in samples exposed to both constant higher temperature and oscillating temperatures for 14 and 28 days **(**Fig. 1D**)**.

### Increased temperature increases the predominance of ARG-harboring subpopulations

**Fig. 2:**
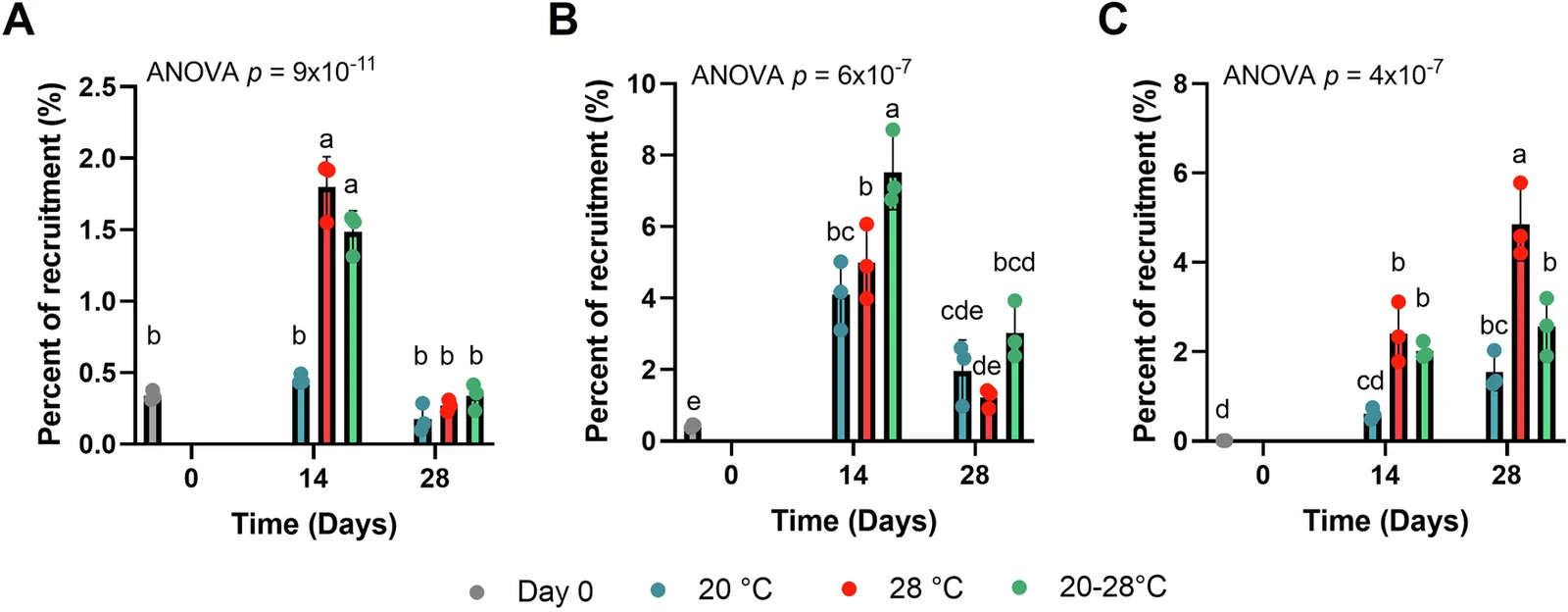
Relative abundance of the three MAGs containing antibiotic resistance genes selected at increased temperatures. **A** *Limnohabitans*, completion 60%, redundancy 4%, contains a *pmrE* polymyxin resistance gene. **B** *Limnohabitans*, completion 75%, redundancy 3%, contains a *pmrE* gene. **C** UBA 1122 (Alphaproteobacteria), completion 99%, redundancy 0%, contains a *sul2* sulfonamide resistance gene. Relative abundance was calculated using the percentage of recruitment of each MAG across samples, which represents the percentage of sequences from a sample recruited into a MAG. Error bars represent standard deviation. A *p*-value lower than 0.05 was identified as significant, and the compact letter display was used to represent the significance of Tukey tests. Letters indicate statistical groupings based on pairwise comparisons. Groups that share at least one letter are not significantly different from each other, whereas groups with different letters are significantly different. 20 °C = room temperature controls (blue). 28 °C = constant temperature increase (red). 20–28 °C = repeated temperature increases (green). *n* = 3.

Constant higher temperature and oscillating temperatures selected for several members of the bacterial communities in Rhône river microcosms after 14 and 28 incubation days (Supplementary Table 1 in Supplementary Information), from which three MAGs containing ARGs were identified **(**Fig. 2**)**. Two of the MAGs containing ARGs were annotated as *Limnohabitans* and contained a *pmrE* gene involved in polymyxin resistance. The first one was selected by both constant higher temperature and oscillating temperatures on day 14 **(**Fig. 2A**)**, whereas the second one was selected only by oscillating temperatures on day 14 **(**Fig. 2B**)**. On the other hand, a third *Limnohabitans* MAG with no identified ARGs showed a decay in relative abundance under all conditions over time (Supplementary Fig. 3A in Supplementary Information). Therefore, different temperature regimes appeared to induce changes in bacterial subpopulation dynamics. The third MAG that contained an ARG was annotated as Alphaproteobacteria and had a *sul2* sulfonamide resistance gene. This MAG was selected by both the constant higher temperature and oscillating temperatures on day 14 and by the constant higher temperature on day 28 **(**Fig. 2C**)**. Although most MAGs containing specific ARGs were selected by at least one kind of temperature increase at one incubation time, a fourth MAG associated with *Panacagrimonas* that contained an *aac(3’)-Ia* gentamicin resistance gene was not selected by any increased temperature regime (Supplementary Fig. 3B in Supplementary Information). Whereas the relative abundance of the three ARG-containing MAGs was increased under exposure to increased temperatures, their mean coverage did not significantly change across conditions (Supplementary Fig. 4 in Supplementary Information). Thus, the selection of these subpopulations is likely due to their increased predominance resulting from the reduced abundance of other community members, rather than from an increase in their own abundance.

**Fig. 3:**
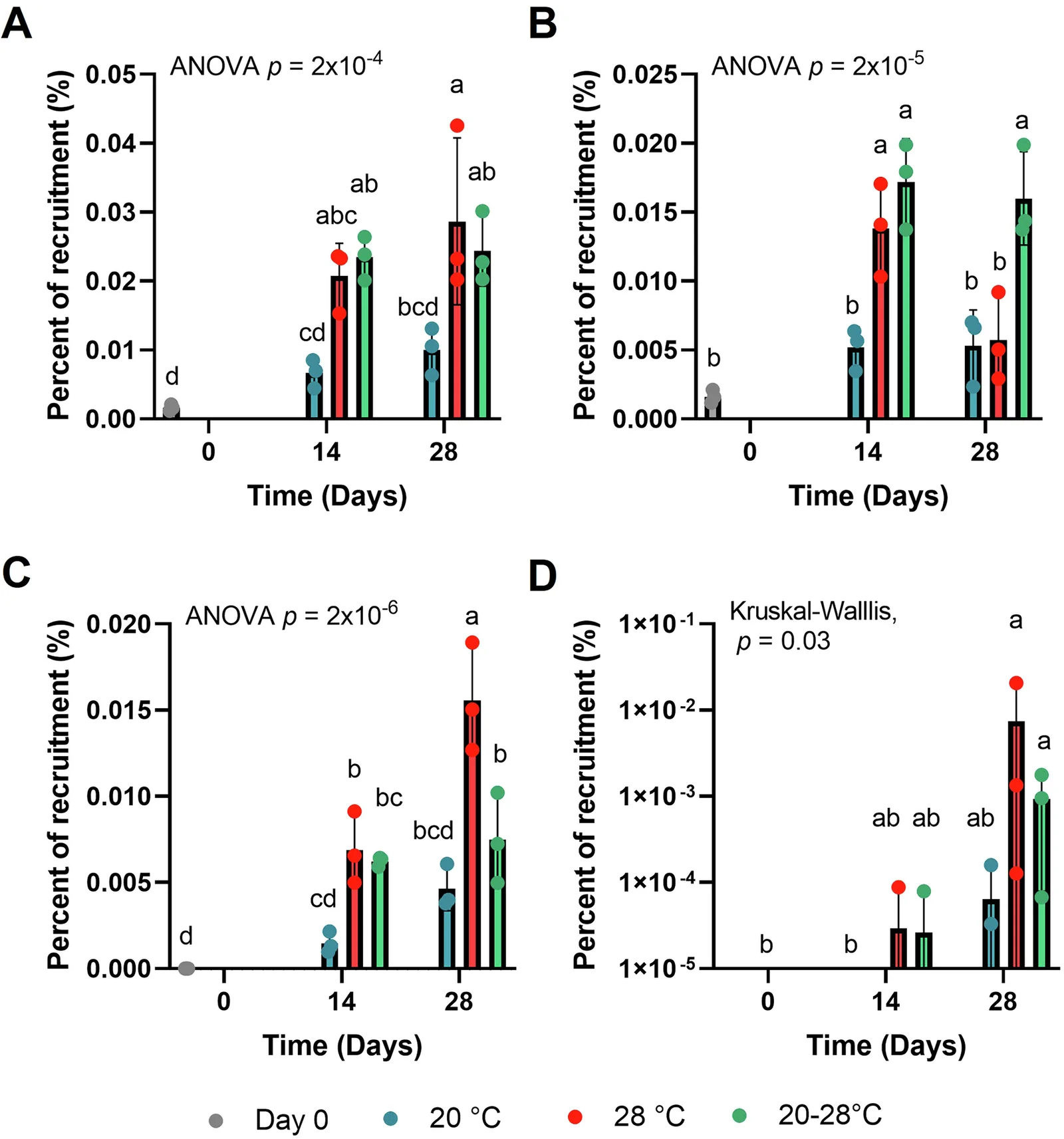
Relative abundance of ARG-containing contigs. All contigs that contain any of the three ARGs identified in the contigs (**A**), contigs that contain the *pmrE* gene (**B**), the *sul2* gene (**C**) and the *aac(3’)-Ia* gene (**D**). Relative abundance was calculated using the percentage of recruitment of each contig across samples, which represents the percentage of sequences from a sample recruited into a contig. Error bars represent standard deviation. A *p*-value lower than 0.05 was identified as significant, and the compact letter display was used to represent the significance of Tukey (**A**–**C**) and Dunn’s (**D**) tests. Letters indicate statistical groupings based on pairwise comparisons. Groups that share at least one letter are not significantly different from each other, whereas groups with different letters are significantly different. 20 °C = room temperature controls (blue). 28 °C = constant temperature increase (red). 20–28 °C = repeated temperature increases (green). *n* = 3.

Since the MAGs contained a minor quantity of contigs, the entire contig pool was examined. Increased temperature regimes in Rhône river microcosms increased the relative abundance of contigs containing ARGs **(**Fig. 3A**):** this abundance was higher in samples exposed to oscillating temperatures for 14 days and in samples exposed to a constant increased temperature for 28 days. When each specific gene was analyzed individually, an increased relative abundance of contigs containing *sul2* was observed in all samples exposed to both increased temperature regimes for 14 days and in samples exposed to oscillating temperatures for 28 days **(**Fig. 3B**)**. The relative abundance of contigs containing *pmrE* also increased in samples exposed to a constant temperature increase for 14 and 28 days **(**Fig. 3C**)**. On the other hand, no significant increases were found in the relative abundance of contigs containing *aac(3’)-Ia* **(**Fig. 3D**)**, MRGs and genes related to MGE (Supplementary Fig. 4C in Supplementary Information).

### Oscillating temperatures alter the aquatic microbiome transcriptional response

**Fig. 4:**
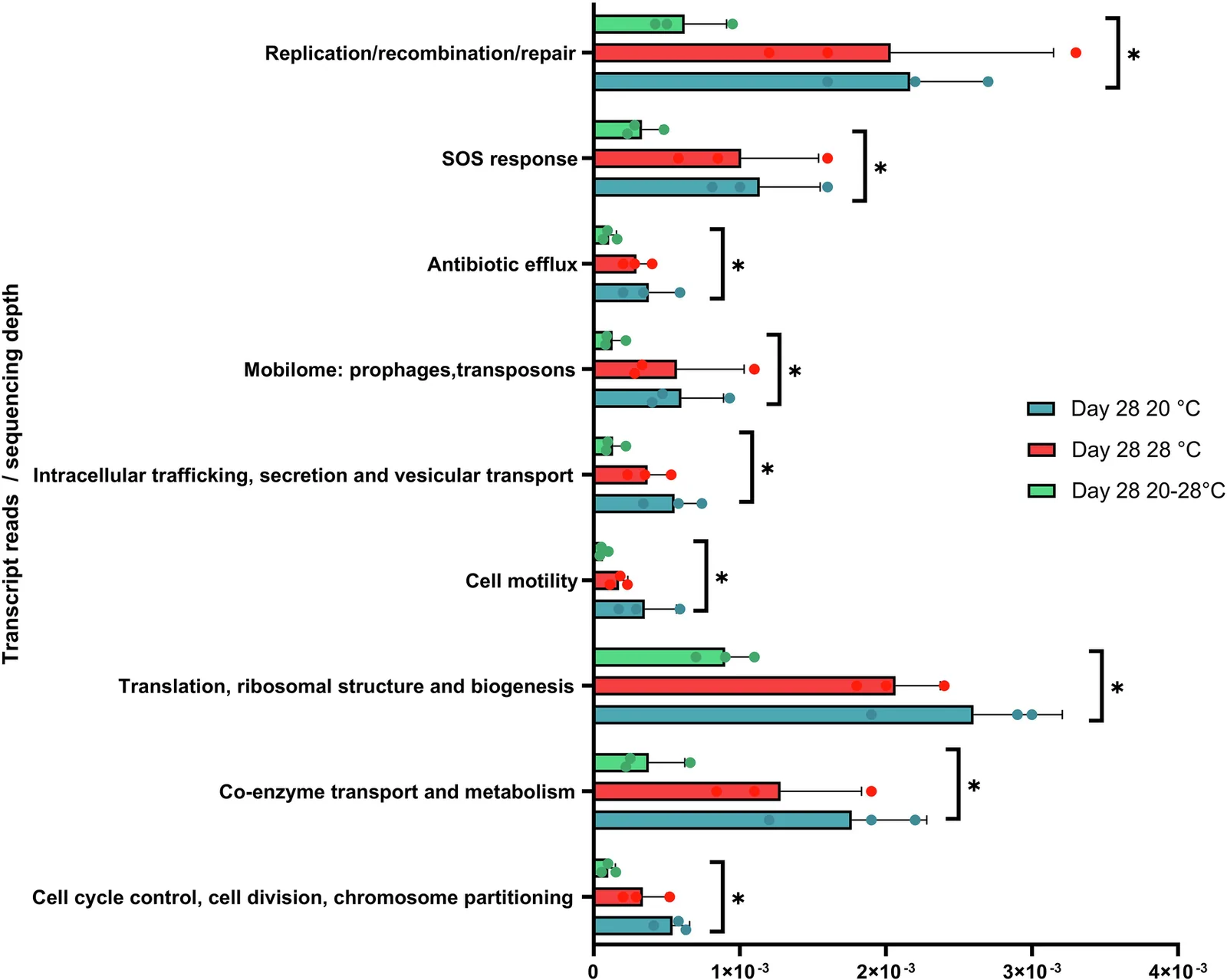
Differences in transcript relative abundance across conditions after 28 days. Transcript abundances obtained from metatranscriptomic sequences are normalized by sequencing depth. Transcripts were identified using multiple databases (mobileOG for replication/recombination/repair, a custom Uniprot database for SOS response, CARD for antibiotic efflux, and COG for intracellular trafficking, secretion and vesicular transport; cell motility; translation, ribosomal structure and biogenesis; co-enzyme transport and metabolism; cell cycle control, cell division, chromosome partitioning). Error bars represent standard deviation. 20 °C = room temperature controls (blue). 28 °C = constant temperature increase (red). 20-28°C = repeated temperature increases (green). A *p*-value lower than 0.05 was identified as significant (*) using Tukey tests for most variables, except SOS response and mobilome, which did not follow a normal distribution and were analyzed using Dunn’s tests. Significant differences were found only between room temperature controls and repeated temperature increases. *n* = *3*.

Finally, bacterial functions affected by increased temperatures were identified using metatranscriptomics. On day 14, no differences were found between the relative abundance of transcripts associated with bacterial physiology, stress responses, antibiotic resistance, and gene mobility across conditions (Supplementary Fig. 5 in Supplementary Information). On the other hand, transcripts associated with several processes linked to cell division, bacterial activity and metabolism, cell motility, as well as antibiotic efflux, gene mobility and stress responses showed a lower abundance in samples exposed to oscillating temperatures than in room temperature controls after 28 days **(**Fig. 4**)**. However, the relative abundance of ARG transcripts was similar across conditions at all exposure times (Supplementary Fig. 6 in Supplementary Information).

## Discussion

The aim of this study was to determine whether increases in river water temperature select for antibiotic resistance genes and to identify the bacterial responses that take place under increased temperatures in a model aquatic ecosystem. This research shows that increased river water temperature altered bacterial community composition and selected for bacterial subpopulations that contained ARGs. In addition, oscillating temperatures (between 20 and 28°C) reduced the transcription of several processes linked to bacterial metabolic activity, cell division, cell motility, antibiotic efflux, gene mobility, and stress responses over 28 days.

Increased and/or oscillating river water temperatures shaped bacterial communities in river water microcosms (Fig. 1) and showed a selective impact on river water bacteria, selecting for some populations that carry ARGs. The role of temperature as an abiotic factor shaping bacterial community composition has been widely studied in a variety of ecosystems, including the human microbiome^18^, the cryosphere^19^, wastewater^20^ and soil^21^. Our results show specific and distinct communities being selected under constant increased and oscillating temperatures as well as at different incubation times (Fig. 2). The identification of several MAGs belonging to *Limnohabitans* that show different dynamics under increased temperatures over time highlights how some bacterial sub-populations may be better adapted to increased and/or frequently variant temperatures, which might help them outcompete other sub-populations from the same genus. *Limnohabitans* is a Proteobacterium commonly found in the environment. It has been previously related to antibiotic resistance selection when the same aquatic ecosystem, the Rhône River, was exposed to low levels of anthropogenic pressure^22^. The only *Limnohabitans* MAG that does not contain ARGs was the only one showing a reduced relative abundance in samples exposed to increased temperatures. Although this raises questions about the potential role that ARGs could play in the selection of their hosts, an increase in transcription levels of the ARGs encoded by the MAGs selected under temperature increases was not observed. Furthermore, the dynamics of the two *Limnohabitans* MAGs that contain the same gene are heterogeneous (Fig. 2A-B), and temperature increases had a stronger impact on the composition of bacterial communities than on that of the antibiotic resistome (Fig. 1). These observations suggest that other physiological traits may be driving these changes at the subpopulation level. Further research should explore temperature-driven dynamics of aquatic bacteria at the species level to better understand the mechanisms that underlie the heterogeneous selection of subpopulations belonging to the same genus.

ARGs were selected in samples exposed to constant increased and oscillating water temperatures (Fig. 3), whereas this was not the case for antibiotic efflux pump genes and metal resistance genes. These results are consistent with previous research showing increased levels of antibiotic resistance at warmer temperatures in water^23^, biofilms^24^, aquacultures^25^ and fish^26^. Although temperature increases selected for bacteria carrying ARGs, not all bacteria selected at warmer temperatures carried these genes, and some bacteria that contained ARGs were not selected under temperature increases, as illustrated by the *Panacagrimonas* MAG that had an aminoglycoside resistance gene. In addition, contrary to previous findings showing that warmer environments may increase HGT rates^17^ and the abundance of some mobile genetic elements (MGE)^26^, no measurable changes were observed in the abundance and transcription of genes related to MGE and HGT, except for the reduced transcription of prophage and transposon-associated genes under oscillating temperature conditions. These findings, as well as the heterogeneity in the dynamics of individual ARGs across time and conditions and the similar transcription levels of these specific ARGs across conditions, suggest that the selection of antibiotic resistance genes induced by constant higher temperature and oscillating temperatures is indirect. The indirect role of thermal disturbance on the antibiotic resistome through the shaping of bacterial communities has previously been observed in soil^27^. Antibiotic resistance gene selection may be collateral damage of the selection of populations that tolerate increased temperatures, rather than a specific process induced by increased temperatures. Therefore, we propose thermal disturbance as a possible indirect driver of antibiotic resistance gene maintenance in aquatic ecosystems through the selection of some ARG-containing bacteria that are better adapted to higher and/or varying temperatures.

Regarding gene transcription, oscillating temperatures reduced the abundance of transcripts involved in several processes related to bacterial activity, stress responses, cell division, antibiotic efflux, cell and gene mobility (Fig. 4). One possible explanation is that the repeated changes in water temperature lead to sensitive members of the community entering a reversible dormant state^28^. Consequently, due to the higher availability of nutrients, non-sensitive members of the community could dominate and maintain their activity during the periods where sensitive members are dormant, which is consistent with the observed shifts in community composition and the similar ribosome numbers across conditions. Previous studies have shown the impact of increased temperatures on the induction of bacterial dormancy^29,30^. On the other hand, in samples exposed to a constant temperature increase over the whole experiment, sensitive members entering dormancy would not have the opportunity to revert to this state. Thus, non-sensitive members of the community would thrive over the 28 days, inducing shifts in bacterial community composition while keeping gene transcription patterns similar to controls. Positive relationships have been found between functional redundancy and water temperature^31^, and previous studies have found significant changes in community composition under temperature increases with a conserved functional profile^32,33^. Thus, the two stresses applied in this study induced different medium-term responses in the community, and although both of them translated into a selection of bacteria carrying ARGs, there are some differences in the selected members and the mechanisms involved.

Bacteria and genes that show an increased relative abundance under temperature increases are commonly found in river ecosystems. *Limnohabitans* is a widespread freshwater bacterial genus dominant in bacterioplankton that has been identified in several studies on Rhône River bacterial communities^22,34^. However, this environmental genus has not been shown to colonize the human gut. On the other hand, *pmrE* and *sul2* are ubiquitous and have been found in both environmental and human microbiomes. The *pmrE* gene was first described in an enteric pathogen^35^, although it is also found in sediment and seawater microbiomes^36^. The *sul2* gene is ubiquitous and widely distributed,

often detected in river water microbiomes^37^, as well as in the human gut^38^. This gene is the most prevalent determinant of sulfonamide resistance, and it is plasmid-associated^39^, which facilitates its spread across hosts and habitats. Metagenomic assembly is biased towards predominant members of the community. Thus, although the increased predominance of *Limnohabitans* and *UBA 1122* (an uncultured genus of environmental origin) is unlikely to result in a direct risk to human health, the genes hosted by these environmental bacteria collaterally become more predominant in the community too, and they might be horizontally transferred to human microbiome bacteria, including pathogens, upon exposure to river water. The indirect selection of ARGs induced by temperature increase might therefore increase the risk of antibiotic resistance spread from water bodies to human microbiomes. Future research should focus on quantifying the potential human health risk associated with ARG selection in environmental microbiomes exposed to global change.

In summary, this research shows that increases and oscillations in river water temperature may select for bacterial subpopulations carrying ARGs and that different responses are induced by constant increased and oscillating temperature regimes. Thus, this study provides new insights into the disturbance induced by climate change in river water communities. However, this study was focused on antibiotic resistance at a genetic and transcriptional level, and although ARGs are a relevant bioindicator of the evolution of antibiotic resistance under temperature changes, the impact of increased temperatures on phenotypic environmental resistance was out of the scope of this study. Future research should address whether these temperature perturbations select for phenotypic antibiotic resistance in complex aquatic ecosystems and identify the populations that express ARGs under increased temperature scenarios. In addition, this research was done on a single model aquatic ecosystem using a microcosm approach with controlled conditions, and the spatiotemporal heterogeneity of aquatic ecosystems/microbiomes in the world may also impact the response of these bacteria and their associated resistome to thermal perturbations. Microcosm experiments are critical for tracking the evolution of the same original community under stress. However, they have inherent limitations, such as nutrient depletion over long periods, which restrict their applicability for assessing the long-term persistence of selected traits, as well as community adaptation and stabilization. In addition, the complexity of the response induced in natural water bodies is expected to be higher due to additional temperature fluctuations between day and night, inconstant temperature variations over long periods of time covering a wide temperature range, the dynamic nature of riverine ecosystems, and the presence of multiple physical and chemical stressors other than temperature shifts. Thus, further in situ research should investigate how aquatic bacteria respond to thermal stress in multiple geographical locations and aquatic ecosystems that are subject to a range of anthropogenic and environmental pressures. This will complement these findings and improve our understanding of how antibiotic resistance is selected in scenarios of climate change in the environment.

## Methods

### River water sampling and microcosm set-up

Rhône river water was sampled from the surface on January 30th 2024, in Lyon (45°45'08.1“N 4°50'11.4“E). Twenty L were sampled, and samples were processed within 24 hours. Two L river water microcosms named room temperature controls were prepared and incubated at 20 °C, the lowest water temperature observed in the Rhône in August 2023, one of the warmest months of that year (Supplementary Fig. 7 in Supplementary Information). In parallel, 2 L microcosms were incubated at 28 °C (constant thermal disturbance) and under repeated thermal disturbances (intervals of 3 days at 20 °C and 4 days at 28 °C) for 28 days to determine the medium-term impact of thermal disturbance on the maintenance of antibiotic resistance. A temperature of 28 °C has already been observed on the surface of the Rhône River during periods of extreme heat (Supplementary Fig. 7 in Supplementary Information). Three microcosms were prepared for each temperature regime. Samples were shaken at 40 rpm to mimic river agitation. DNA and RNA-based analyses were performed on samples at day 0 before the beginning of the experiment and after 14 and 28 incubation days at different temperature treatments (*n* = 3).

### DNA/RNA extraction and 16S rRNA gene/cDNA quantitative qPCR

One hundred mL of river water was filtered per sample through a 0.2 µm pore size filter. DNA and RNA were co-extracted from the filters following the Phenol/Chloroform method^40^. DNA was eluted in 40 µl of nuclease-free water and conserved at −20°C. RNA was eluted in 20 µl of nuclease-free water, treated with RQ1 RNase-Free DNase (Promega), and conserved at −80°C. The abundance of the bacterial communities in river water was estimated by quantifying the 16S rRNA gene by qPCR using the 341F (5′-CCTACGGGAGGCAGCAG- 3′) and 534R (5′-ATTACCGCGGCTGCTGGCA-3′) primers^41^. The activity of bacterial communities was estimated by quantifying 16S rRNA from cDNA retrotranscribed using the SuperScript III Reverse Transcriptase (Invitrogen) using 341F and 534R primers. qPCR amplification was carried out using the CFX Duet Real-Time PCR System (Bio-Rad) in a 20 µl reaction volume containing QuantiNova SYBR Green PCR Master Mix (Qiagen), 0.75 µM of each primer, and 2 µl of DNA. Since 16S rRNA is involved in translation in bacteria, it can be used as a proxy for overall bacterial activity in complex communities. Two non-template controls were included in the assay, as well as one retrotranscription control. The standard curve was obtained using 10-fold serial dilutions of a linearized plasmid pGEM-T Easy Vector (10^7^–10^2^ copies) containing the 16S rRNA gene of *Pseudomonas aeruginosa* PAO1. Cycling conditions for qPCR amplification were 95 °C for 2 min followed by 35 cycles of 95 °C for 5 s and 60 °C for 30 s. Melt curves were generated by increasing temperature from 60 °C to 95 °C after amplification. The reaction had an efficiency of 100% and a linearity R^2^ coefficient of 0.98.

### Metagenomic sequencing

Metagenomes were sequenced from DNA extracted from river water samples. Metagenomics libraries were prepared from ≤1 ng of DNA using the Nextera XT Library Prep Kit and Indexes (Illumina) as detailed in Illumina’s “Nextera XT DNA Library Prep Kit” reference guide. DNA sequencing was performed using the NextSeq 1000 System and the NextSeq 1000/2000 P2 Reagent Kit 600 cycles (Illumina). Reads were quality-filtered according to the criteria described by Minoche et al.^42^. Sequencing depths after quality control are shown in Supplementary Table 2 in Supplementary Information. Quality-filtered short reads were co-assembled (all samples were assembled together) using MEGAHIT^43^ and reads were mapped onto the >1 kb contigs obtained from the assembly using Bowtie2^44^. The assembled contigs were blasted against the CARD antibiotic resistance gene (ARG) database^45^, the BacMet metal resistance gene (MRG) database^46^ and the mobileOG mobile genetic element (MGE) database^47^ using Diamond^48^, which translates nucleotide queries prior to blasting, in order to determine ARG, MRG, and MGE relative abundance in the assembled contigs. The results obtained from the contig-based analysis were filtered at a minimum amino acid identity of 60%, a minimum length of 150 amino acids, and a maximum e-value of 10^−5^. The best hit was chosen (highest-scoring remaining hit per query based on bitscore). Gene abundance was represented as the percentage of recruitment (percentage of reads from a sample that are recruited in each ARG/MRG/MGE-containing contig) and is thus normalized by sequencing depth.

Then, profiles were created for each individual sample and merged using the anvi’o^49^ metagenomic workflow (version 8). Contigs were binned based on their differential coverage across samples using anvi’o, and the bins were refined based on differential coverage and sequence composition. Bins with <50% completion and ≥10% redundancy were discarded, since they were considered low-quality metagenome assembled genome (MAG) drafts^50^. MAG taxonomy was estimated using Centrifuge^51^, integrated in the anvi’o workflow. The number and length of assembled contigs, as well as the number of total and high-quality MAGs ( ≥ 95% completion and <5% redundancy) obtained from water metagenomes, are shown in Supplementary Table 3 in Supplementary Information. MAG length, GC content, completion and redundancy are shown in Supplementary Table 4 in Supplementary Information.

### Metatranscriptomic sequencing

Metatranscriptomes were sequenced from RNA samples extracted from river water. Double-stranded cDNA was generated from DNase-treated RNA using the NEBNext Ultra II Directional RNA Synthesis Module (New England Biolabs). Then, metatranscriptomic libraries were prepared and sequenced following the protocol used for metagenomic libraries. Reads were quality-filtered according to the criteria described by Minoche et al. and treated using fastp to remove artificial “G” created by the sequencer when transcripts were shorter than 300 bp (average read length was 97 bp). Sequencing depths after quality control and treatment are shown in Supplementary Table 4 in Supplementary Information. Quality-filtered short reads were blasted against the CARD ARG database, the mobileOG MGE database, and two custom-made databases from Uniprot sequences related to SOS response and heat shock response genes using Diamond to identify gene transcripts that could be involved in the response to temperature increases according to previous studies. In addition, reads were blasted against the Cluster of Orthologous Genes (COG20) database^52^ to classify gene transcripts in major families involved in bacterial physiology. The obtained results were filtered at a minimum amino acid identity of 60%, a minimum length of 25 amino acids, and a maximum e-value of 10^−5^. The best hit was chosen, and transcript abundance was normalized by sequencing depth.

### Statistical analyses

Statistical analyses were performed using R Studio v4.3.231, and graphics were generated using Prism GraphPad v 9.5.0. Normality was tested using the Shapiro-Wilk test. ANOVA and post-hoc Tukey tests were used to determine statistical differences between normally distributed variables, whereas Kruskal-Wallis and post-hoc Dunn’s tests were used on variables that did not follow a normal distribution. A *p*-value lower than 0.05 was identified as significant, and the compact letter display was used to represent the significance of Tukey/Dunn’s tests^53^.

Non-metric Multidimensional Scaling (NMDS) analyses were performed to evaluate the impact of increased temperatures on community composition and the aquatic resistome and mobilome. Bray-Curtis distances were calculated from the relative abundance of MAGs (total, ARG-containing, and MRG-containing) and ARG, MRG, and MGE-containing contigs, and NMDS analyses were done using the “vegan” package in R. PERMANOVA tests with 999 permutations were applied to each NMDS to determine the significance of the impact of increased temperatures on the composition of bacterial communities, the antibiotic and metal resistome, and the mobilome using the “adonis2” package in R.

## Supplementary information

Supplementary Information

## Data Availability

The datasets generated and analyzed during the current study are available at the DDBJ repository, BioProjects PRJDB19656 and PRJDB20410 (DRA accession DRA019744).

## Code availability

All codes used in this study are available at: https://github.com/concscid/Bousquet-et-al.
