# Supplementary material for: Increased temperature selects for antibiotic resistance genes in a model aquatic ecosystem
Source: NPJ Antimicrob Resist. 2026 Jun 12;4:66. doi: 10.1038/s44259-026-00231-6 (PMC13661515; doi:10.1038/s44259-026-00231-6)
Supplement: Supplementary file 1 [file 44259_2026_231_MOESM1_ESM.pdf]

Supplementary Information

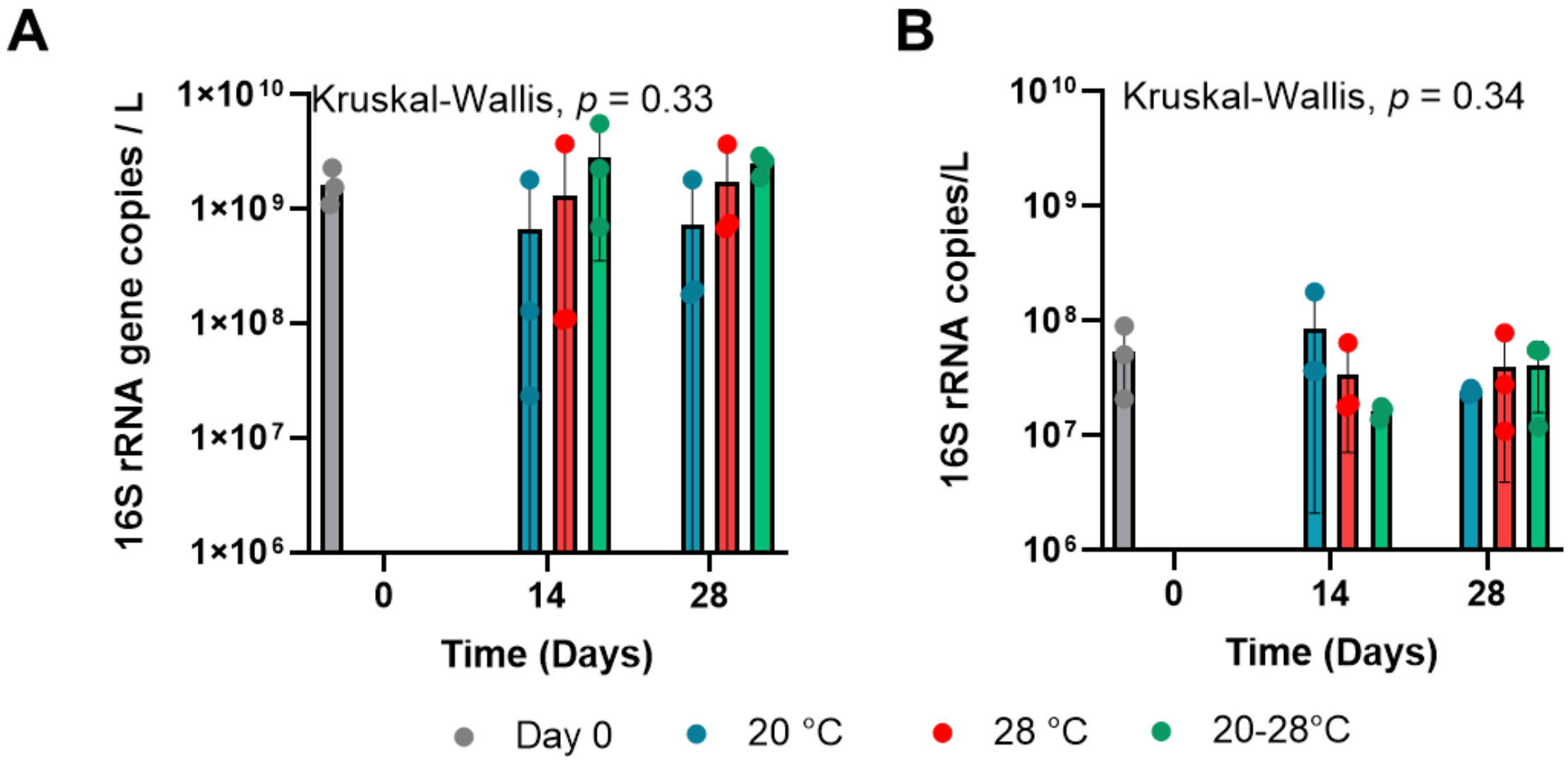

Supplementary Figure 1. **Impact of constant and repeated thermal disturbances on overall bacterial abundance and activity.** Overall bacterial abundance was measured by ribosomal gene counts (A) and transcriptional activity was measured by ribosomal counts (B)*.* Error bars represent standard deviation. 20°C = room temperature controls (blue). 28°C = constant temperature increase (red). 20-28°C = repeated temperature increases (green). *n*=3.

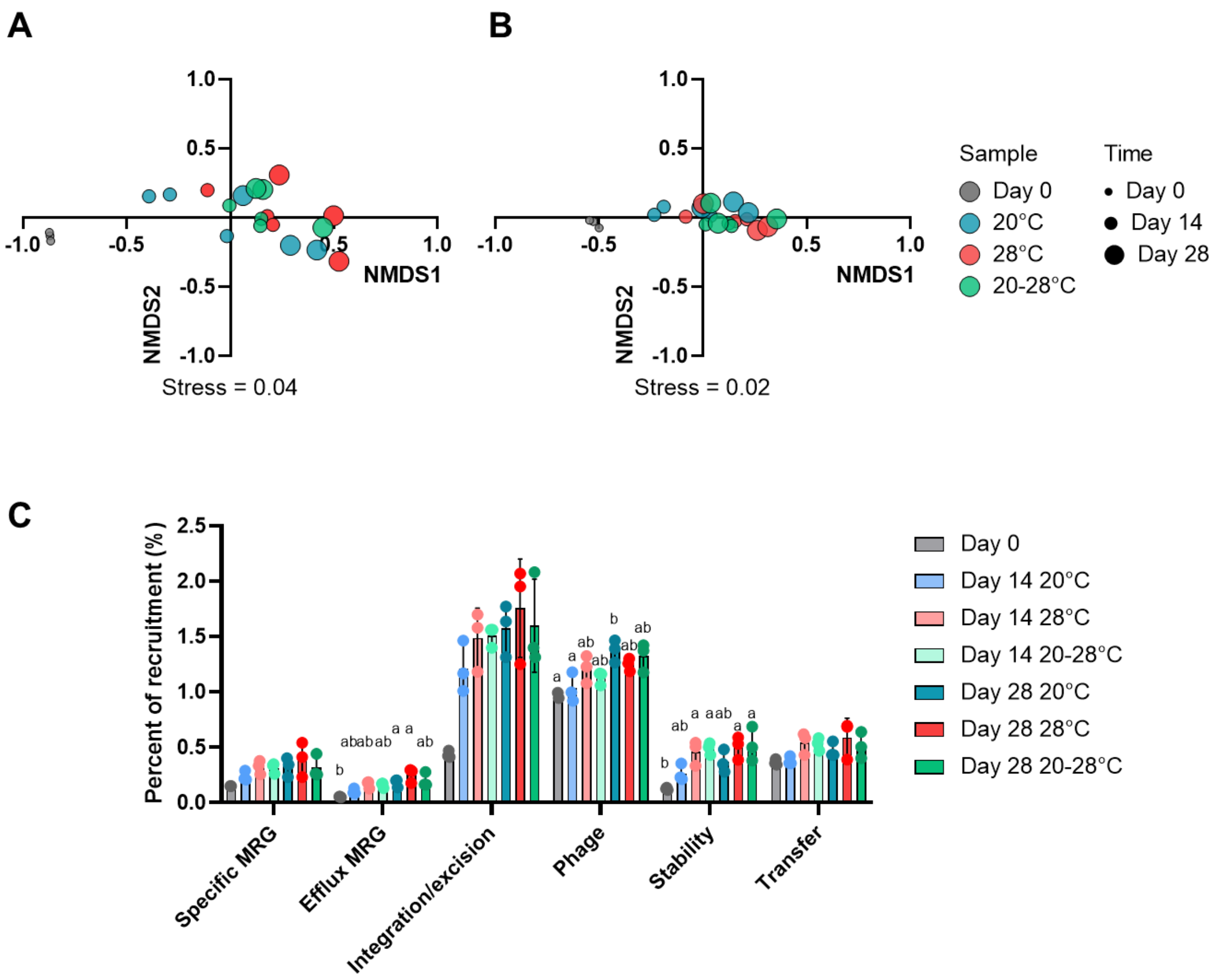

Supplementary Figure 2. **Impact of increased temperature on the metal resistome and the mobilome. A:** Non-metric multidimensional scaling of the Bray-Curtis distances calculated from the relative abundance of contigs that contain metal resistance genes. **B:** Non-metric multidimensional scaling of the Bray-Curtis distances calculated from the relative abundance of contigs that contain mobile genetic elements. **C:** relative abundance of the sum of contigs containing specific and generalist metal resistance genes, and genes related to mobile genetic elements. Error bars represent standard deviations; *n*=3. A: PERMANOVA without day 0 R^2=0.15 F=1.5 *p*=0.23. B: PERMANOVA without day 0: R^2=0.19 F=2 *p*=0.16. C: A *p*-value lower than 0.05 was identified as significant, and the compact letter display was used to represent the significance of Tukey tests. Each category was compared independently; when all conditions belonged to the same group (a), comparisons are not shown. 20°C = room temperature controls (blue). 28°C = constant temperature increase (red). 20-28°C = repeated temperature increases (green).

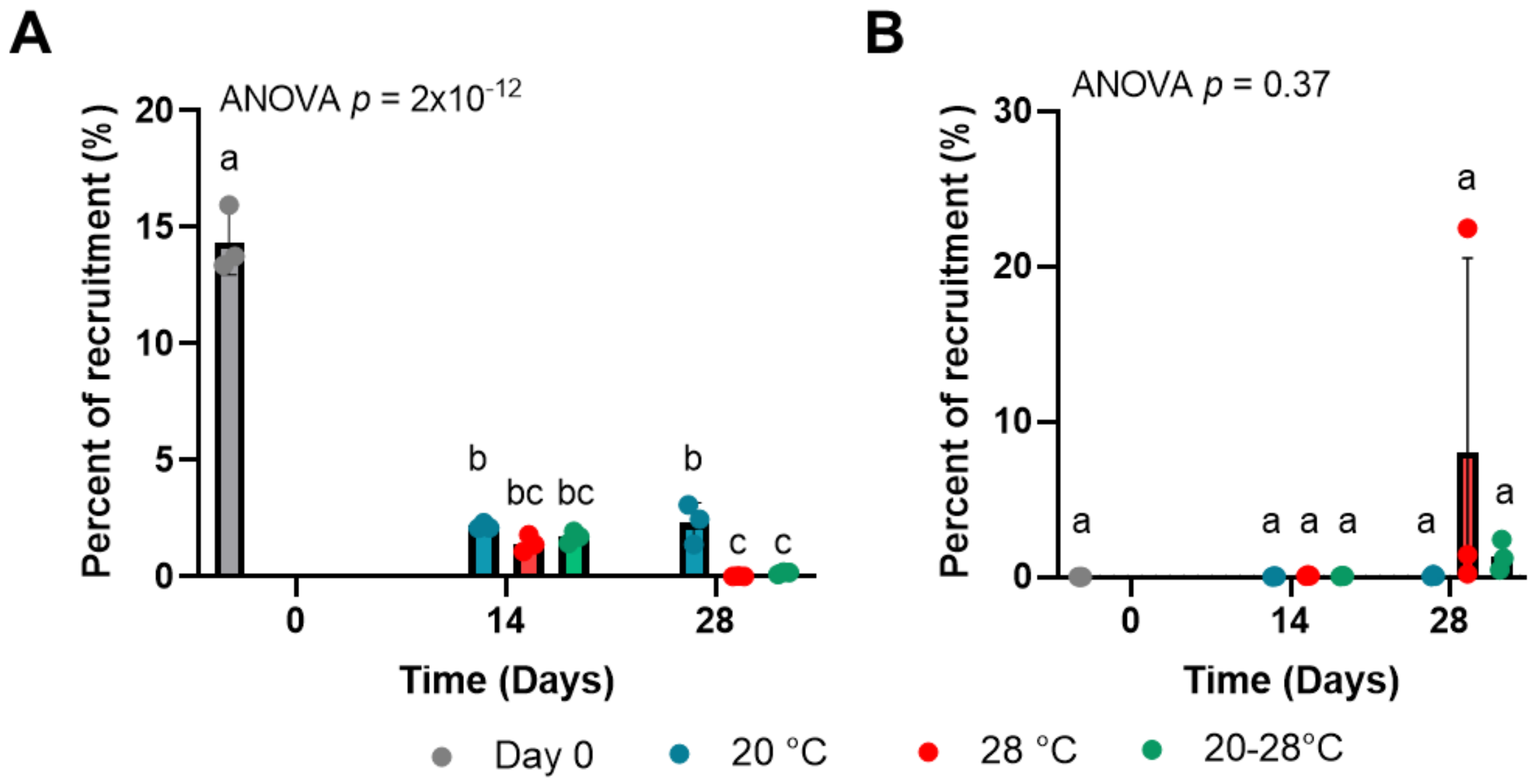

Supplementary Figure 3. Relative abundance of a *Limnohabitans* MAG without any identified ARG and a *Panacagrimonas* MAG containing an *aac(3')-Ia* gentamicin resistance gene. A: *Limnohabitans, c*ompletion 69%, redundancy 9.8%. **B:** *Panacagrimonas, c*ompletion 76%, redundancy 3%. Error bars represent standard deviation. Relative abundance was calculated using the percentage of recruitment of each MAG across samples, which represents the percentage of sequences from a sample recruited into a MAG. 20°C = 20°C = room temperature controls (blue). 28°C = constant temperature increase (red). 20-28°C = repeated temperature increases (green). *n*=3.

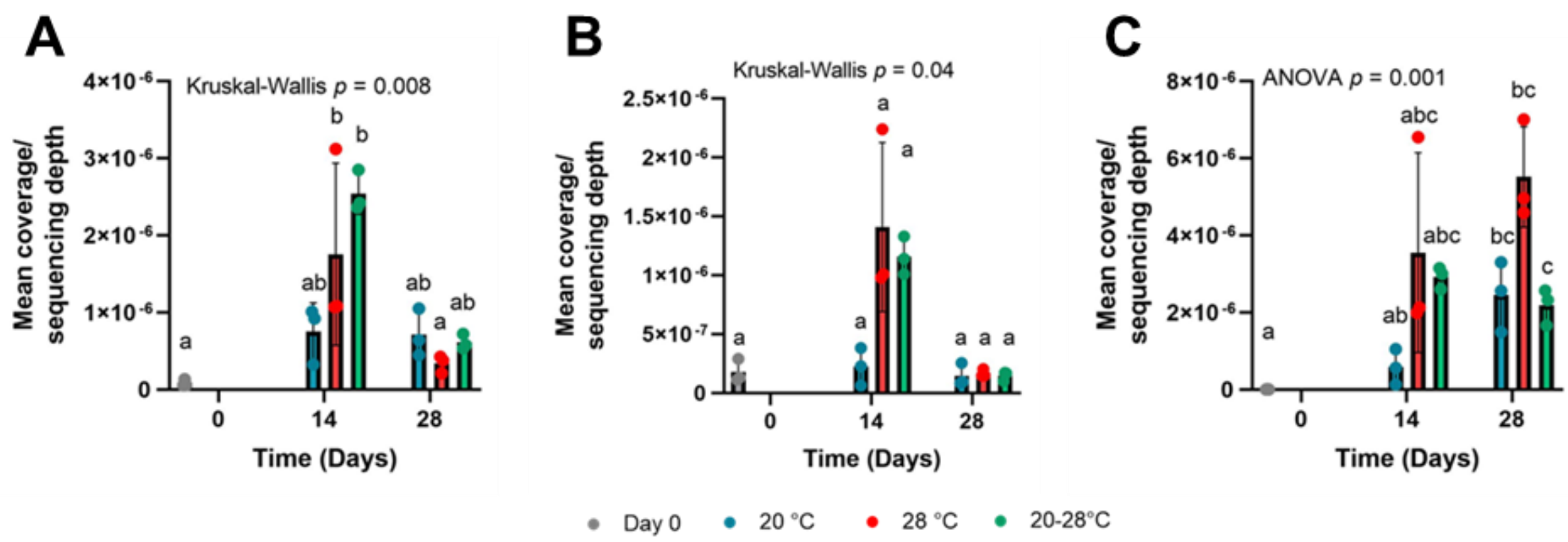

Supplementary Figure 4. **Mean coverage normalized by sequencing depth of the three MAGs containing antibiotic resistance genes selected at increased temperatures.** A: Limnohabitans, completion 60%, redundancy 4%, contains a pmrE polymyxin resistance gene. B: Limnohabitans, completion 75%, redundancy 3%, contains a pmrE gene. C: UBA 1122 (Alphaproteobacteria), completion 99%, redundancy 0%, contains a sul2 sulfonamide resistance gene. Error bars represent standard deviation. A p-value lower than 0.05 was identified as significant, and the compact letter display was used to represent the significance of Tukey tests. Letters indicate statistical groupings based on pairwise comparisons. Groups that share at least one letter are not significantly different from each other, whereas groups with different letters are significantly different. 20°C = room temperature controls (blue). 28°C = constant temperature increase (red). 20-28°C = repeated temperature increases (green). *n*=3.

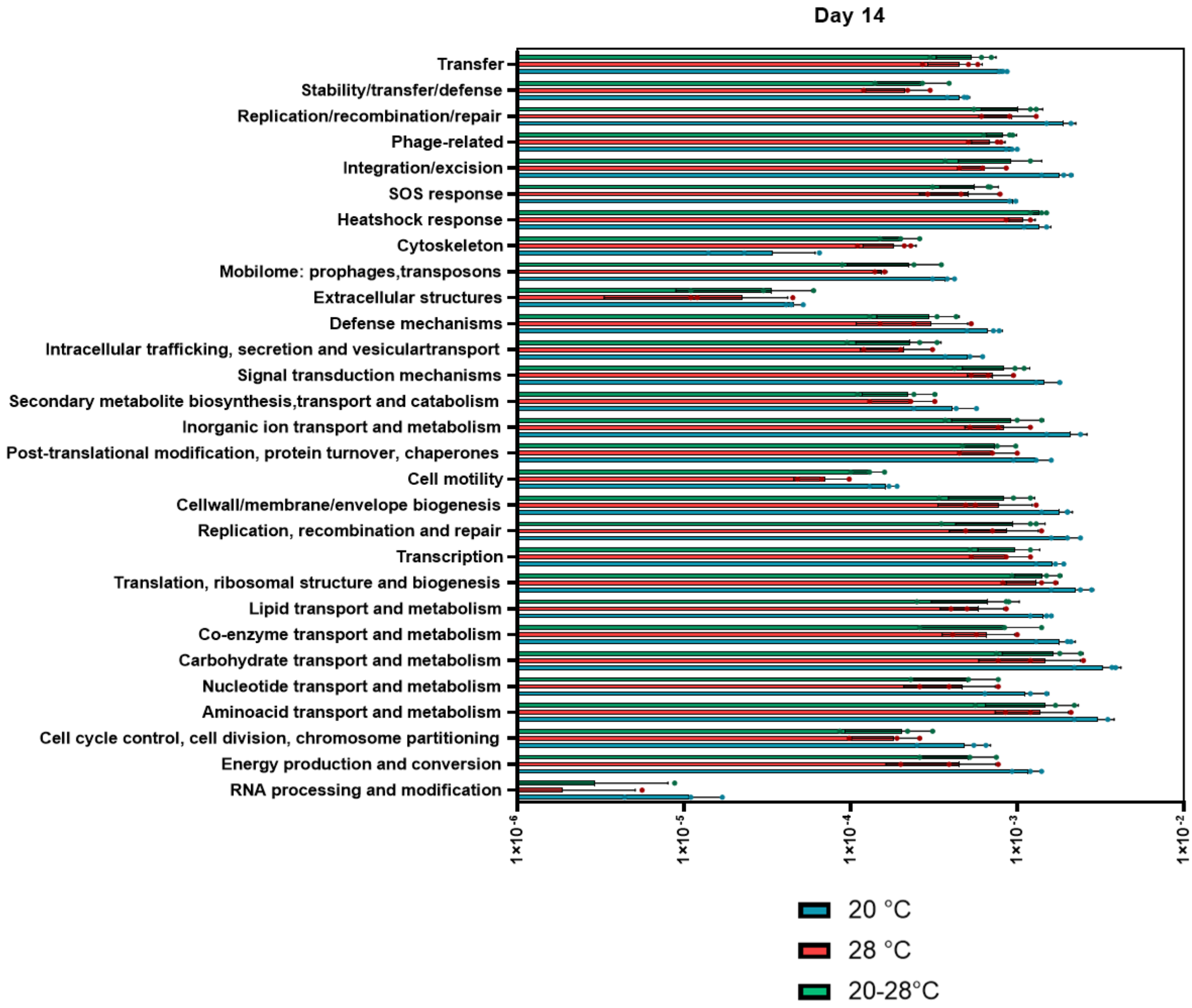

Supplementary Figure 5. **Transcript relative abundance across conditions after 14 days.** Transcript abundances obtained from metatranscriptomic sequences are normalized by sequencing depth. Transcripts were identified using multiple databases (mobileOG for replication/recombination/repair, a custom Uniprot database for SOS response, CARD for antibiotic efflux and COG for intracellular trafficking, secretion and vesicular transport; cell motility; translation, ribosomal structure and biogenesis; co-enzyme transport and metabolism; cell cycle control, cell division, chromosome partitioning). Error bars represent standard deviation. No significant differences were found. 20°C = room temperature controls (blue). 28°C = constant temperature increase (red). 20-28°C = repeated temperature increases (green). *n*=3.

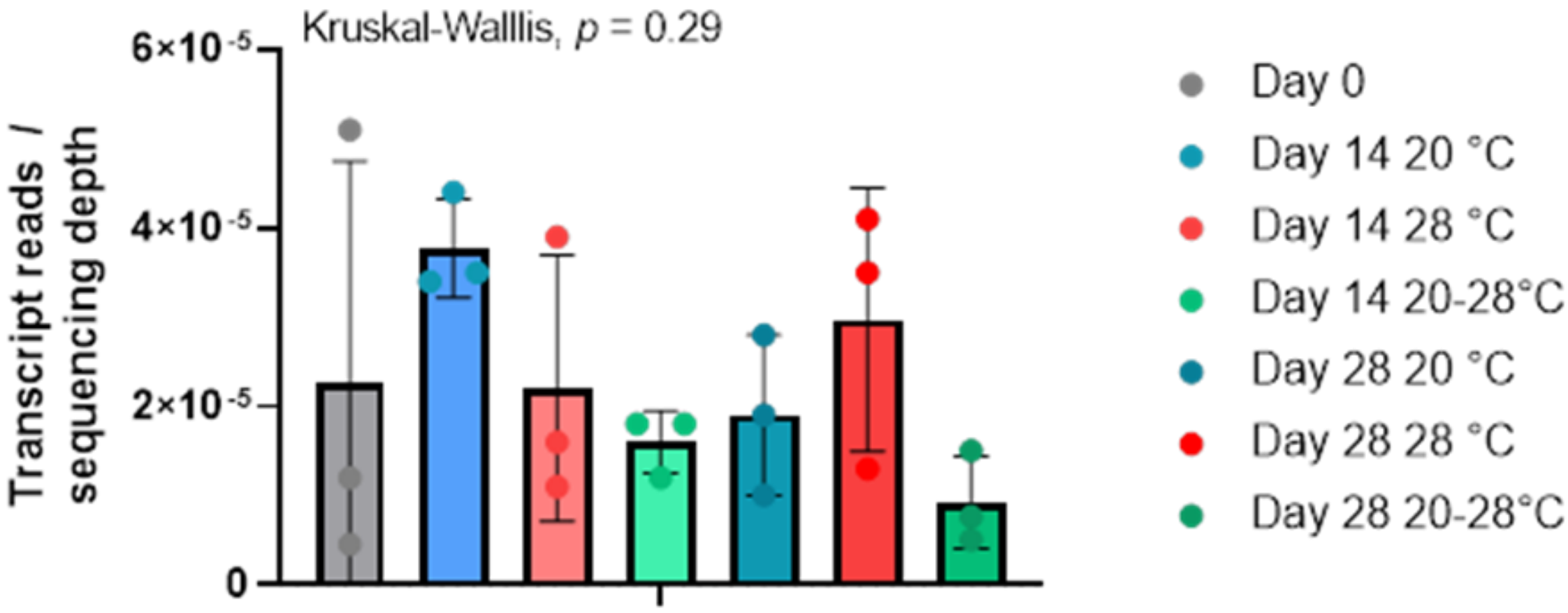

Supplementary Figure 6. Relative abundance of transcripts associated to ARGs identified in the metatranscriptomic sequences of Rhône river water samples. Transcript abundance was normalized by sequencing depth. Error bars represent standard deviation. 20°C = room temperature controls (blue). 28°C = constant temperature increase (red). 20-28°C = repeated temperature increases (green). *n*=3.

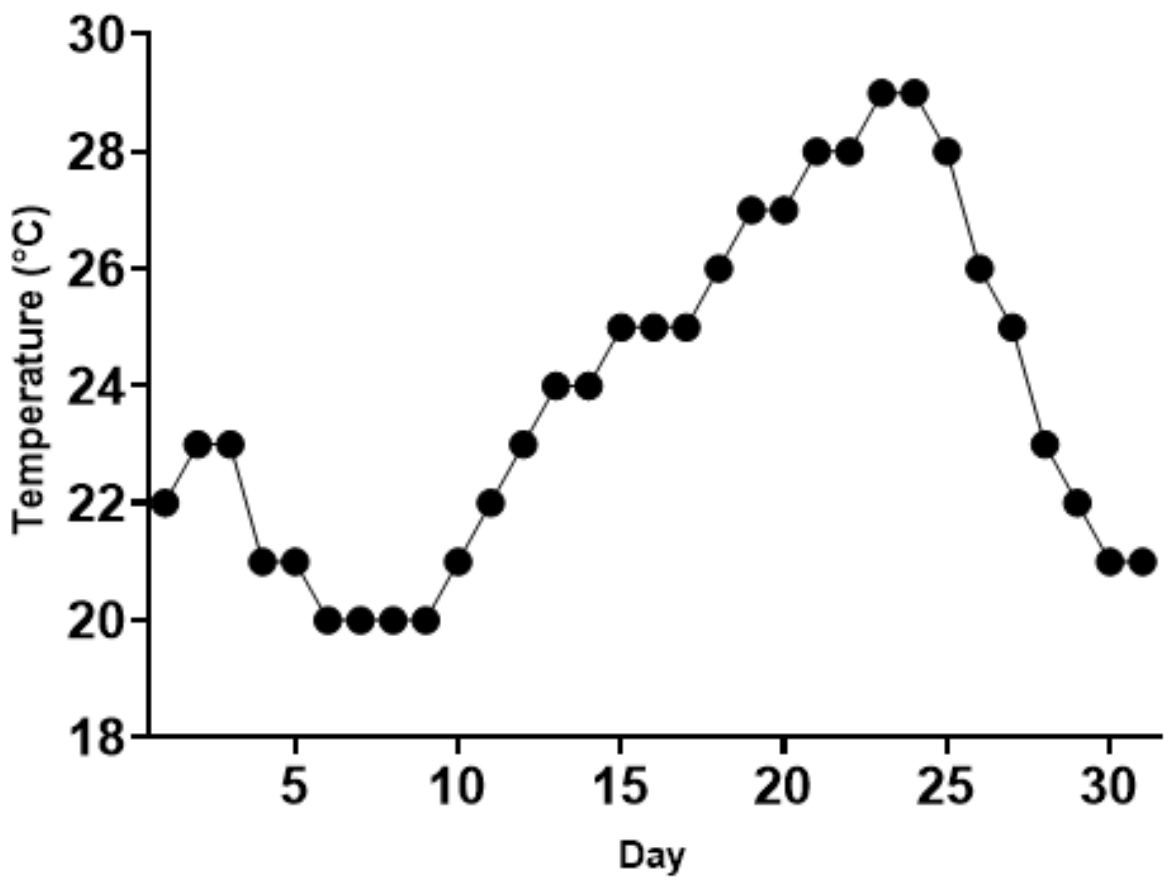

Supplementary Figure 7. Daily temperature of Rhône surface water in Lyon during August 2023 (Source: www.eaufrance.fr).

Supplementary Table 1. Taxonomy, completion, redundancy and ARG content of other MAGs selected by increased temperature.

Phylum	Genus	Completion (%)	Redundancy (%)	Antibiotic efflux pumps	ARGs	Selected by
Pseudomonadota	*Ga0077536*	100	3	*oqxB*	-	28°C (day 14)
Verrucomicrobiota	*SXTU01*	94	7	*mexB*	-	28°C (day 14)
Patescibacteria	*C7867*	65	1	-	-	28°C, 20-28°C (day 28)
Actinomycetota	*BACL27*	58	6	-	-	28°C, 20-28°C (day 28)
Pseudomonadota	*NA*	82	9	-	-	28°C (day 28), 20-28°C (days 14 and 28)
Actinomycetota	*F1-60-MAGs027*	65	6	-	-	20-28°C (day 28)
Bacteroidota	*UBA955*	61	1	-	-	20-28°C (day 28)
Bacteroidota	UBA952	97	1	-	-	28°C, 20-28°C (day 14)
Planctomycetota	SXKJ01	77	1	-	-	20-28°C (day 14)
Planctomycetota	*SYIB01*	96	3	-		20-28°C (day 28)
Bacillota	*Copromonas*	86	4	-	-	20-28°C (day 28)

Supplementary Table 2. Number of metagenomic sequences obtained from water DNA samples after quality control. Triplicates for each condition are separated by commas.

Temperature \ Day	20°C	28°C	20-28°C
**Day 0**	3607040, 1676082, 3952097	No samples	No samples
**Day 14**	3787967, 1037275, 2269774	3707130, 2033984, 2530631	3069844, 2997503, 3010893
**Day 28**	4299252, 2046179, 2977069	1707179, 1571742, 2852257	1667005, 2574265, 2051749

Supplementary Table 3. Summary of the assembly and MAG reconstruction from water metagenomes.

Number of contigs	Average contig length (bp)	Maximum contig length (bp)	Number of MAGs	Number of high-quality MAGs
70317	2447	354894	43	8

Supplementary Table 4. Number of metatranscriptomics sequences obtained from water DNA samples after quality control. Triplicates for each condition are separated by commas.

Temperature \ Day	20°C	28°C	20-28°C
**Day 0**	178028, 400354, 223194	No samples	No samples
**Day 14**	229652, 435260, 172770	178656, 490872, 563360	844308, 499608, 453144
**Day 28**	196998, 324224, 145370	531676, 535420, 338826	888798, 528036, 1206144
